# LncRNA EBLN3P attributes methotrexate resistance in osteosarcoma cells through miR-200a-3p/O-GlcNAc transferase pathway

**DOI:** 10.1186/s13018-022-03449-y

**Published:** 2022-12-21

**Authors:** Ming-Xia Sun, Hai-Yan An, Yan-Bin Sun, Yan-bao Sun, Bing Bai

**Affiliations:** 1 The Operation Room, Chengde Central Hospital, Hebei, China; 2 Department of Anesthesiology, Chengde Central Hospital, Hebei, China; 3 Department of Orthopaedics, Chengde Central Hospital, No. 11 Guangren Street, Shuangqiao District, Chengde, 067000 Hebei China

**Keywords:** LncRNA EBLN3P, Methotrexate, Osteosarcoma, miR-200a-3p, O-GlcNAc transferase

## Abstract

**Background:**

Osteosarcoma is highly malignant. The migration, invasion, and chemoresistance contribute to poor prognosis of osteosarcoma. Research reported that endogenous bornavirus-like nucleoprotein 3 pseudogene (EBLN3P) promotes the progression of osteosarcoma.

**Methods:**

In this study, the expression of EBLN3P in osteosarcoma tissue with different methotrexate (MTX) treatment responses was measured. Osteosarcoma cell lines with MTX resistance were constructed, and bioinformatic analysis was performed to explore the potential involved targets and pathways.

**Results:**

Higher EBLN3P was associated with MTX resistance. Downregulation of LncEBLN3P decreased the MTX resistance of osteosarcoma cells by sponging miR-200a-3p, an important microRNA that affects epithelial-mesenchymal transition (EMT). The decreased miR-200a-3p resulted in the upregulation of its target gene O-GlcNAc transferase (OGT), which in turn promoted the EMT process of osteosarcoma cells. Further analysis confirmed that the loss of OGT and over-expression of miR-200a-3p could partly abolish the MTX resistance induced by LncEBLN3P.

**Conclusion:**

LncEBLN3P is upregulated in osteosarcoma and increases the MTX resistance in osteosarcoma cells through downregulating miR-200a-3p, which in turn promoted the EMT process of osteosarcoma cells by increasing the OGT.

## Introduction

Osteosarcoma is one of the malignant orthopedic tumors that occur in young under the age of 30. The advance in neoadjuvant chemotherapy increases the 5-year overall survival rate by over 60% [1]. Currently, the commonly used treatment is surgery plus chemotherapy. However, the resistance to chemotherapy affects the curative effect and prognosis of osteosarcoma [2]. The treatment of new adjuvant chemotherapy drugs and immunotherapy has not achieved great breakthroughs yet [3], and it is particularly important to find the molecular targets involved in drug resistance to osteosarcoma. The commonly used reagents for chemotherapy include cisplatin, methotrexate (MTX), ifosfamide, and doxorubicin [4]. Increasing the dose intensity of MTX during chemotherapy can improve patients' 5-year tumor-free survival rate, but high-dose MTX has potential toxic side effects and even induces drug resistance [5]. Metastasis and chemotherapy resistance become the main factors restricting the overall survival rate in patients with osteosarcoma.

LncRNA is a group of noncoding RNA with a length of more than 200 bp. The transcription of lncRNA is generated from independent promoters by RNA polymerase II. They participate in the progression of various cancers and drug resistance. As an important epigenetic regulator, LncRNAs mainly regulate the expression of related functional proteins via miRNA [6]. And the miRNA-based therapies have great potential compared with traditional therapies for diseases treatment, including musculoskeletal diseases [7–9]. LncEBLN3P is a pseudogene of endogenous bornavirus-like nucleoprotein 3 (EBLN3) located on chromosome 9. Research has demonstrated that EBLN3P is over-expressed in tumors [10–12], including osteosarcoma [13].

Increased O-GlcNAc transferase (OGT) expression has been reported in many human cancers [14]. A domestic study in China reported the expression of OGT in osteosarcoma tissues increased and downregulating its expression increased the sensitivity of osteosarcoma cells to cisplatin [15]. Deng et al. [16] reported that OGT degradation contributed to tumor necrosis factor-related apoptosis-inducing ligand (TRAIL) resistance. The epithelial-to-mesenchymal transition (EMT) increases the motility of cancer cells. EMT also plays important role in maintaining stemness properties, which contributes to the chemoresistance of cancer cells [17]. Loss of miR-200 expression is correlated with the EMT process of tumors [18, 19]. An in vitro analysis indicated that overexpression miR-200a could inhibit osteosarcoma cell proliferation, promotes apoptosis, and increases cellular radiosensitivity [20]. In this study, we used clinical specimens, further with the help of in vitro molecular methods, upregulation or knocking-down LcnEBLN3P expression in osteosarcoma cells, to investigate the biofunction of LncEBLN3P in invasion, migration, and MTX resistance of osteosarcoma cells, and finally explored whether it play its function via the miR-200a-3p/O-GlcNAc transferase signaling axis.

## Methods

### Osteosarcoma tissue samples and cell lines

The osteosarcoma tissues and the adjacent non-tumor tissues were obtained from the surgical resection sample of our hospital. The freshly resected tissues were preserved at − 80 °C refrigerators or fixed for pathological examination. All patients were diagnosed with osteosarcoma by pathological examination and did not receive any anti-tumor treatment before the operation. The informed consent was obtained from each patient and the study was approved by the ethics committee of our hospital.

In this study, patients who received cisplatin + ifosfamide + high dose methotrexate regimen after the operation and had preserved samples were screened. Patients who received at least 2 cycles of treatment were selected. A total of 53 pairs of samples and the medical data of these patients were collected. The sensitivity of methotrexate (MTX) was classified by the treatment response: MTX sensitive group (complete response or partial response, and the maintenance time ≥ 4 weeks), and MTX-resistant group (stable disease or progressive disease) according to the Response Evaluation Criteria in Solid Tumors (RECIST) [21].

### Immunohistochemistry

Fresh tissue samples were fixed with 4% neutral paraformaldehyde buffer, routinely dehydrated, waxed, embedded, and then cut into a 4-µm slide. Paraffin sections were baked, dewaxed, and antigen repaired with sodium citrate for 20 min. The slide was sealed with bovine serum albumin (BSA), and OGT antibody (#PA5-83154, Invitrogen, Foster, USA) diluted at 1:50 was dropped and incubated overnight in a 4 °C refrigerator. Rabbit secondary antibody (#PA5-59497) was added and incubated at room temperature for 1 h. Diaminobenzidine (DAB) was used to develop color, hematoxylin was used for counterstain. The slide was sealed with neutral gum and observed and photographed under an optical microscope (× 400 magnification, Olympus, Japan).

### Induction of MTX-resistant osteosarcoma cells

Human osteosarcoma cell lines MNNG/HOS, Saos-2, U2OS, and MG63 were purchased from cell bank of the Chinese Academy of Sciences, Shanghai. MTX-resistant cells were inducted through gradually increasing MTX concentration. Briefly, MTX with an initial concentration of 50 ng/ml was added to the logarithmic proliferating osteosarcoma cells. After treated for 24 h, cells were washed and cultured with McCoy's 5A (#16600082, Thermo Fisher Scientific, USA) or DMEM medium (#10566016, Thermo Fisher Scientific) without drugs to restore the normal proliferation state and repeat the same concentration until the cells can adapt to this concentration and escalated to the next concentration. The increased concentrations of MTX were 50 ng/ml, 100 ng/ml, 200 ng/ml, 500 ng/ml, 1000 ng/ml, and 2000 ng/ml. The stably growing MTX (2000 ng/ml)-resistant cell lines were termed MTXr, such as MNNG/HOS-MTXr.

### Measurement of MTX sensitivity in cells

The logarithmic growth cells were digested with 0.25% trypsin, resuspended, and prepared into single-cell suspension with culture medium. A total of 200 µL cells with a concentration of 5 × 10^4^/ml were inoculated into 96 well plates and cultured for 24 h. MTX with gradient concentrations (including one control well) were prepared according to the pre-experiment results. Three multiple wells were set for each concentration. After cultured for 24 h, 20 µL MTT (#C0009M, Beyotime, China) were added to each well and cultured for another 4 h. The optical density (OD) of each well at 490 nm was detected by an enzyme labeling instrument to calculate the cell viability. Cell viability (%) = OD of experimental group / OD of control group × 100%. Detailed MTX concentration in osteosarcoma cells was: MNNG/HOS: 0, 1, 3, 4, 5 µM; MNNG/HOS-MTXr: 0, 2, 5, 10, 20 µM; MG63: 0, 1, 2, 3, 4 µM; MG63-MTXr: 0, 2, 5, 10, 20 µM.

### Real-time quantitative PCR (RT-qPCR)

RT-qPCR was used to detect the expression of EBLN3P, miR-200a-3p, and OGT. Briefly, total RNA was extracted from preserved tissues or cultured cells by a commercial kit (#R1200, Solarbio Life Science, Beijing, China). The extracted RNA was reverse transcribed into cDNA. PCR was carried out according to the instructions of the quantitative kit (#RR037A, TaKaRa Bio Inc., Japan) and performed on 7500 Real-Time PCR Systems (Applied Biosystems, Foster, CA, USA). GADPH and U6 were used as internal references, respectively. The relative expression was calculated by the 2(− δ C(T)) method prescribed previously [10]. Used primers are listed in Table 1.

**Table 1:** Primers for RT-qPCR

Gene	Sequence
EBLN3P	F: 5′–GTGTTGTCCCGGAAGTGCC–3′
	R: 5′–TTGAAGGTTTGCCTTCTCTG–3′
miR-200a-3p	F: 5′–TAACACTGTCTGGTAACGATGT–3′
	R: 5′–CATCTTACCGGACAGTGCTGGA–3′
OGT	F: 5′–TTCGGGAATCACCCTACTTCA–3′
	R: 5′–TACCATCATCCGGGCTCAA–3′
GAPDH	F: 5′–TTGATGGCAACAATCTCCAC–3′
	R: 5′–CGTCCCG TAGACAAAATGGT–3′
U6	F: 5′–CTCGCTTCGGCAGCACA–3′
	R: 5′–AACGCTTCACGAATTTGCG–3′

### RNA Oligoribonucleotides and cell transfection

The transfection was performed using Lipofectamine™ 2000, according to the manufacturer’s instructions. Cells were plated in a 24-well plate with a density of 1 × 10^4^ cells per well. The transfected vector includes lentivirus harboring an shRNA of LncRNA EBLN3P (EBLN3P-si), miR-200a-3p mimic, OGT overexpression (OGT OE, #HG17892-NM, Sino Biological Inc., Beijing, China), OGT interfering sequence (OGT-si) and related negative control sequence, respectively. The EBLN3P-si and overexpression of EBLN3P (pcDNA-EBLN3P, EBLN3P OE) were used to knock down and upregulation of LncRNA EBLN3P expression, respectively. The other related sequences used in this study were synthesized by RIBOBIO Co., Ltd. The transfection efficiency was determined by qRT-PCR methods [22].

### Wound healing assay measurement of migration and invasion

Cells with the concentration of 5 × 10^4^ were inoculated into a 6-well plate. After cells were adherent to the culture medium, a sterilized two-milliliter pipette tip was used to generate wounding across the cell monolayer. The debris of cells was washed with PBS twice, and the cells were observed under a microscope at 0, 24, and 48 h after treatment. The scratch width was calculated by ImageJ software (NIH, USA).

### Matrigel invasion assay

Two hundred microliter cells with a concentration of 5 × 10^4^ cells/ml were inoculated into the Transwell chamber (Corning, CA, USA) and then moved into a 24-well plate. Six milliliter mediums containing 10% fetal bovine serum were added to the lower compartment of the chamber and cells were cultured for 48 h. The cells on the compartment of the chamber were fixed with formaldehyde for 20 min and stained with 0.5% crystal violet 20 min. The number of invaded cells in 5 separate fields was counted under the microscope, and the average value was calculated to evaluate the number of invaded cells.

### Microarray data collection

The microarray data used in this study were retrieved from the GEO database (http://www.ncbi.nlm.nih.gov/gds/). The following strategy was used: (Homo sapiens) and (Expression profiling by array) and (Osteosarcoma). We selected data according to the following criteria: Each data set included osteosarcoma sample and normal sample and contained more than 20 samples. GSE65071 data set (plasma from 20 osteosarcoma samples, including 10 localized, 10 metastatic, and 15 healthy controls) and GSE79181 (all came from pre-treatment osteosarcoma tissues, including 9 patients without relapse and 14 patients with relapse) were analyzed. No ethical approval or informed consent was required in this study due to the public availability of data in the GEO databases.

### Dual-luciferase reporter assay

Bioinformatic analysis was performed in online tools TargetScan (http://www.targetscan.org/vert_72/) and ENCORI (https://rna.sysu.edu.cn/encori/agoClipRNA.php?source=mRNA). To verify the binding results in cells, regions containing the binding site of the 3′-UTR targeted were generated by PCR amplification and subcloned into the NheI/XbaI sites of the pmirGLO dual-luciferase reporter plasmid (Cat.#E1330, Promega). The mutations were generated according to the manufacturer’s instructions. The miR-200a-3p mimic or negative control sequence, pmirGLO-EBLN3P-3′UTR plasmid, or mutation plasmid was co-transfected into cells using Lipofectamine 2000 Reagent (Invitrogen). Luciferase activities were measured 48 h after transfection. Firefly luciferase (luc2) was used as the primary reporter to monitor miRNA regulation and Renilla luciferase (hRluc-neo) acted as a control reporter for normalization and selection. Reduced firefly luciferase expression indicates the binding of introduced miRNAs to the cloned mRNA target sequence.

### Western blot analysis

The total protein of tissue homogenate or cells was extracted from lysed cells by a protein extraction kit (#R0010, Solarbio Life Science). A total of 40 µg protein was loaded into each well after protein concentration was determined by BCA test kit (#PC0020, Solarbio Life Science). The proteins were separated by SDS-PAGE and transferred to a nitrocellulose membrane. After being blocked with 5% skimmed milk at room temperature for 120 min, the primary antibody for OGT (#ab250489), Vimentin (#ab52942), N-cadherin (#ab207608), E-cadherin (#ab239883), and GAPDH (#ab9484, all 1:1000 dilution) was added and incubated overnight at 4 °C, then incubated with secondary antibody, and cultured at room temperature for 2 h. ECL developer was loaded, and the expression of the proteins was analyzed by Image J software (NIH, USA).

### Statistical analysis

All data were analyzed by GraphPad 8.0 software. The measurement data were expressed as mean ± standard deviation (SD). The *t*-test or nonparametric test was used to compare values between groups. One-way ANOVA was used for multi-group comparison, and LSD-t-test was used for pairwise comparison. *P* < 0.05 was considered statistically significant.

## Results

### Highly expressed LncRNA EBLN3P is related to methotrexate (MTX) resistance in osteosarcoma

**Fig. 1:**
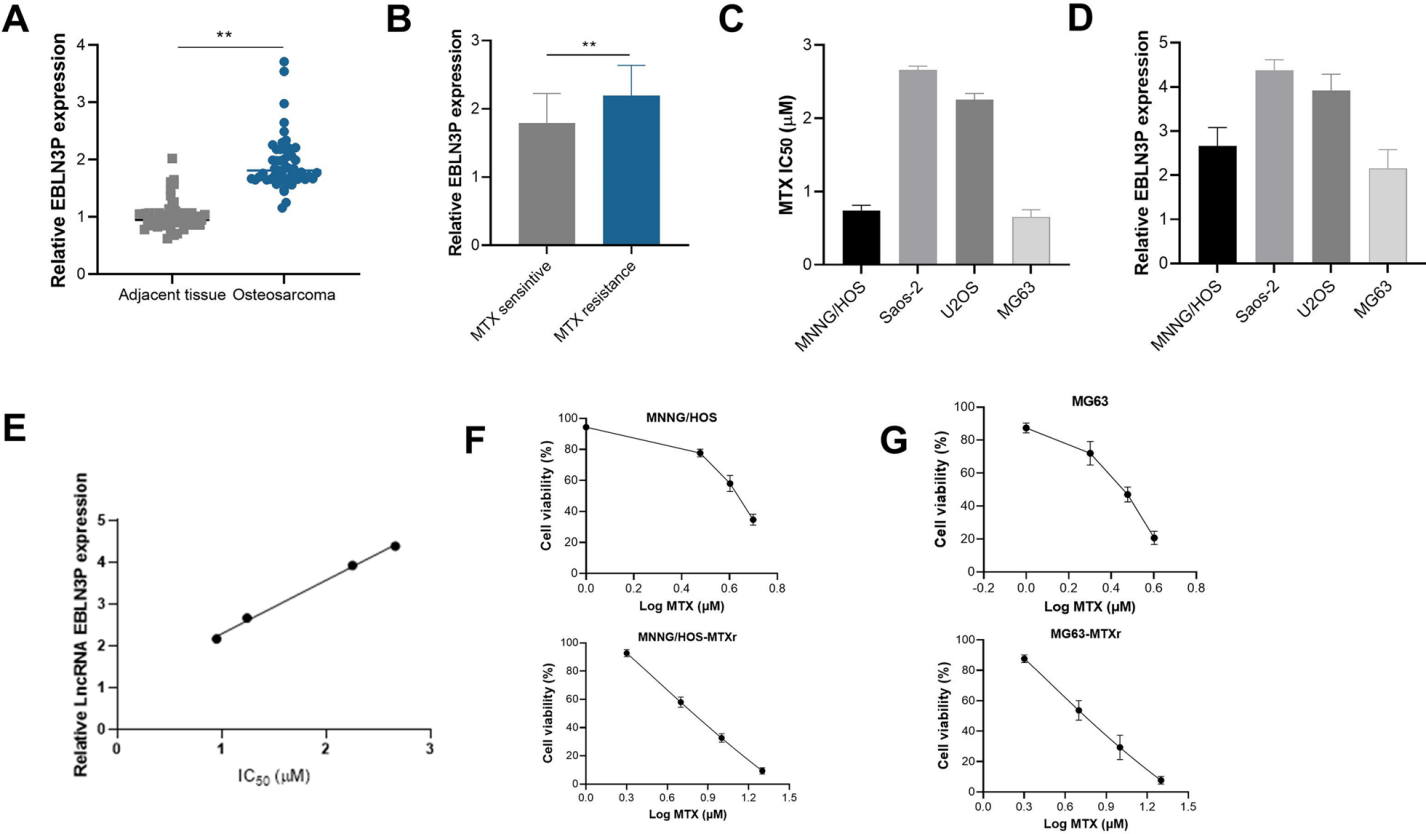
Increased EBLN3P is related to methotrexate (MTX) resistance in osteosarcoma. **A** Expression of EBLN3P in osteosarcoma was significantly higher than adjacent tissues. **B** Patients with MTX resistance have higher EBLN3P expression than MTX-sensitive patients. **C** The IC_50_ of MTX in human osteosarcoma cell line MNNG/HOS, Saos-2, U2OS, and MG63. **D** The expression of LncRNA EBLN3P in each cell lines. **E** The positive correlation of MTX IC_50_ and RNA expression of EBLN3P in each cell lines. **F** and **G** The cell viability of the 2-cell line increased with the induction of MTX, which demonstrated by increased IC_50_ after induction. **p < 0.01

The qRT-PCR analysis showed expression of EBLN3P in osteosarcoma was significantly higher than in adjacent tissues (Fig. 1A). EBLN3P expression in MTX sensitive osteosarcoma tumor tissues was significantly lower than resistant osteosarcoma tumor tissues. (Fig. 1B). Four osteosarcoma cell lines were used in the current study, and their IC_50_ of MTX were screened by MTT assay, which is shown in Fig. 1C. Meanwhile, the expression of LncRNA EBLN3P in each cell lines was also examined by qRT-PCR, which is shown in Fig. 1D. As shown in Fig. 1E, the MTX IC_50_ for each cell lines were positively correlated with RNA expression of EBLN3P, also suggest that higher expression of EBLN3P might be associated with MTX resistance. We selected 2 osteosarcoma cell lines, MNNG/HOS and MG63 with relatively lower EBLN3P expressions used for further MTX-resistant cell establishment, and finally, the MTX-resistant cell line was successfully established, and IC_50_ increased from 0.2 to 1 µM for MNNG/HOS, and 0.35 to 1.3 µM for MG63 (Fig. 1F, G).

### Knockdown EBLN3P increases MTX sensitivity of osteosarcoma cells

**Fig. 2:**
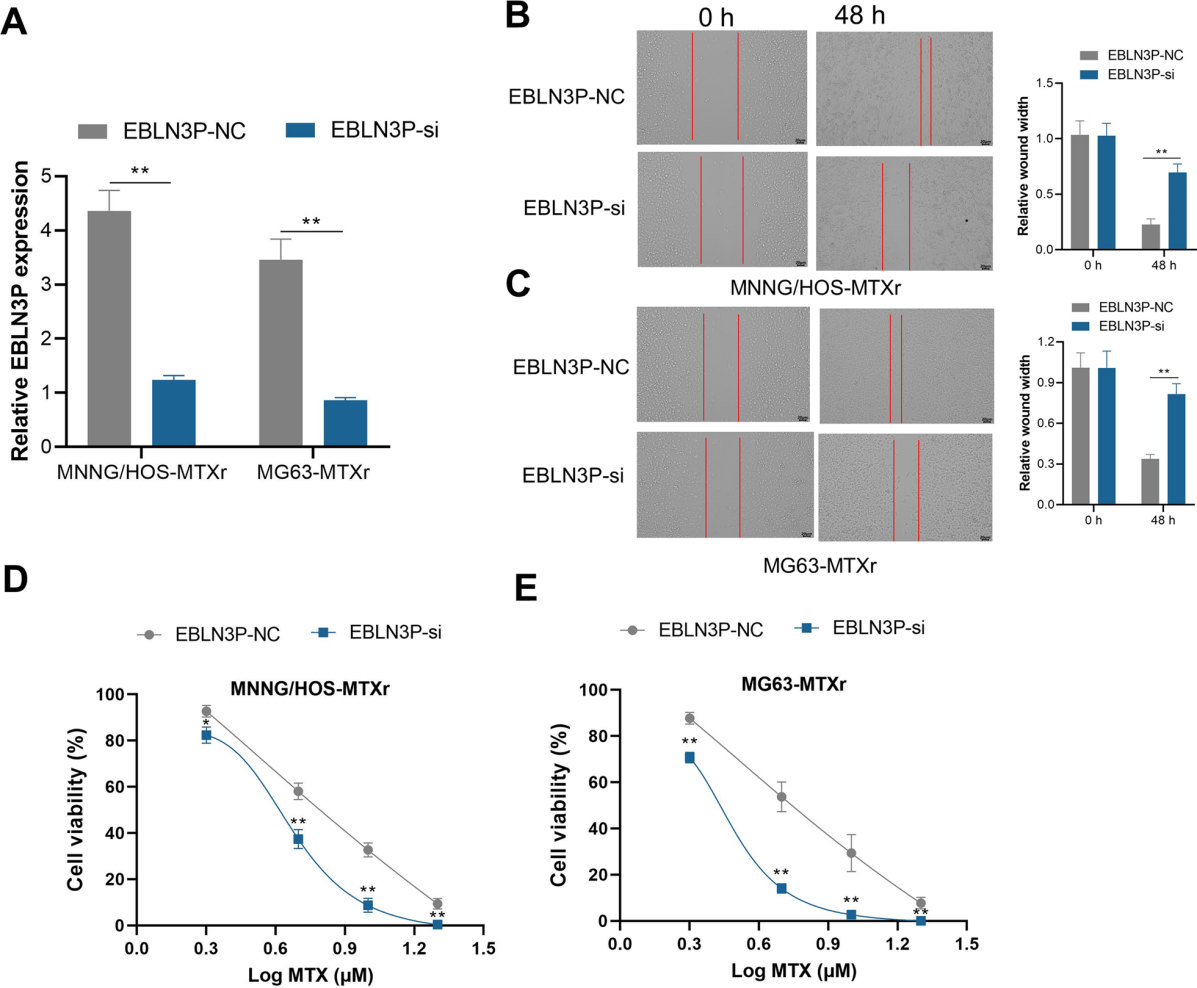
Knockdown EBLN3P increases MTX sensitivity of osteosarcoma cells. **A** qRT-PCR comparison of EBLN3P expression after knocking down EBLN3P (EBLN3P-si). **p < 0.01. **B** and **C** The migration ability of MNNG/HOS-MTXr and MG63-MTXr cells decreased significantly after knockdown of EBLN3P. **p < 0.01. **D** and **E** Comparison of cell viability of the 2 MTX-resistant cell line after knocking down of EBLN3P. * *p *< 0.05, **p < 0.01, compared with EBLN3P NC at the same dose

Three complementary sequences were designed and synthesized to knock down EBLN3P (EBLN3P-si) in osteosarcoma cells, and the sequence with the highest interfering efficacy was used. The interfering efficacy was measured (Fig. 2A). The results showed that knockdown of EBLN3P decreased the migration ability of MNNG/HOS-MTXr and MG63-MTXr cells significantly (Fig. 2B, C). Furthermore, the cell viability of the 2 MTX-resistant cell line decreased after the knocking down of EBLN3P (Fig. 2D, E).

### EBLN3P sponge miR-200a-3p in MTX resistance osteosarcoma

**Fig. 3:**
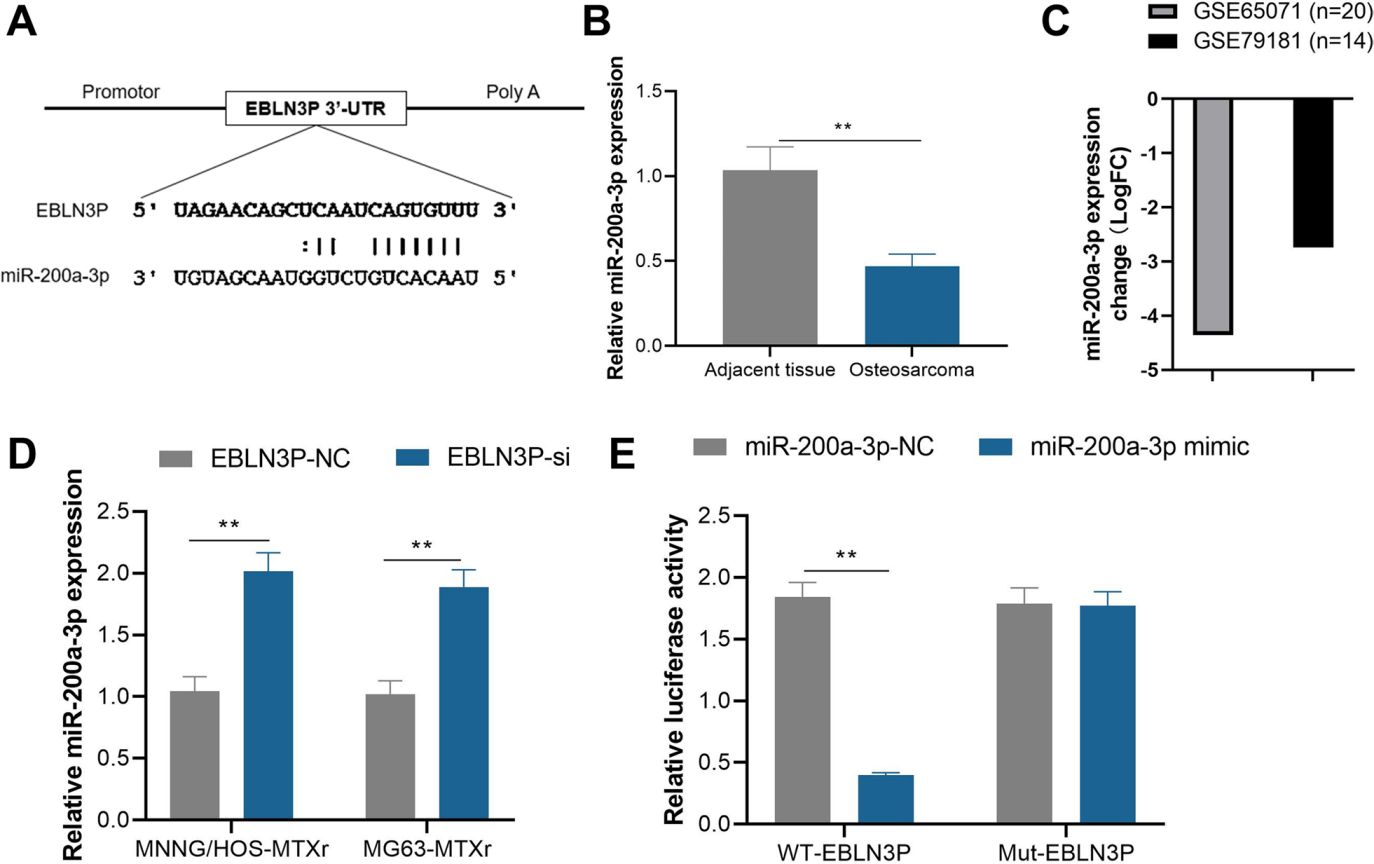
EBLN3P sponge miR-200a-3p in MTX resistance osteosarcoma. **A** The potential binding site of miR-200a-3p with EBLN3P. **B** The expression of miR-200a-3p was significantly decreased in the osteosarcoma tissue compared to adjacent tissues. **C** The expression of miR-200a-3p in osteosarcoma patients of GSE65071 and GSE79181 demonstrated significantly downregulated expression in osteosarcoma patients or osteosarcoma patients with relapse. **D** Knockdown of EBLN3P (EBLN3P-si) increased miR-200a-3p expression in MTX-resistant osteosarcoma cell line MNNG/HOS-MTXr and MG63-MTXr. **E** The luciferase activity of the WT- EBLN3P sequence was suppressed by the mimic of miR-200a-3p while the activity did not affect the Mut- EBLN3P (mutant EBLN3P) sequence compared with negative control sequence of miR-200a-3p (miR-200a-3p NC). **p < 0.01

Bioinformatics analysis demonstrated that miR-200a-3p is one of the potential binding genes with EBLN3P (Fig. 3A). qPCR results demonstrated that the expression of miR-200a-3p was significantly decreased in the osteosarcoma tissue compared to the adjacent tissues (Fig. 3B). To verify our results, the expression of miR-200a-3p in osteosarcoma patients from public data, GSE65071 data set and GSE79181 were analyzed. The results demonstrated expression of miR-200a-3p decreased significantly in osteosarcomas patients and patients with relapse (Fig. 3C). Moreover, knockdown of EBLN3P increased miR-200a-3p expression in MTX-resistant osteosarcoma cells (Fig. 3D). In vitro dual-luciferase activity analysis also demonstrated the luciferase activity of the WT- EBLN3P (wide type EBLN3P) sequence was suppressed by the mimic of miR-200a-3p (miR-200a-3p mimic) while the activity did not affect the Mut- EBLN3P (mutant EBLN3P sequence) sequence compared with negative control sequence of miR-200a-3p (miR-200a-3p NC) (Fig. 3E).

### miR-200a-3p increases MTX sensitivity via O-GlcNAc transferase (OGT)

**Fig. 4:**
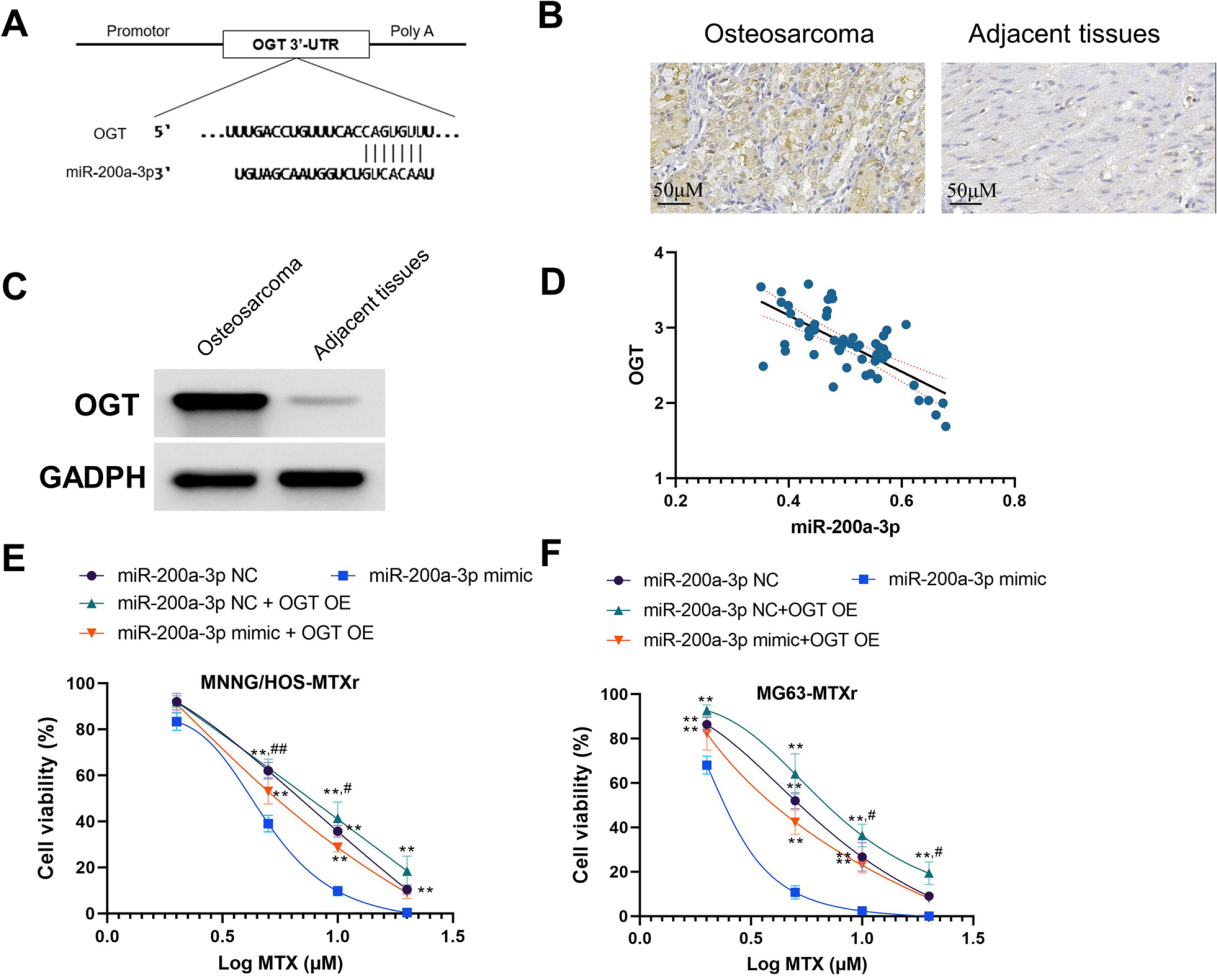
miR-200a-3p increases MTX sensitivity via OGT. **A** The binding site between miR-200a-3p and OGT. **B** and **C** The immunohistochemistry and western analysis of OGT expression in osteosarcoma and adjacent tissues. **D** The correlation analysis showed that the expression of OGT is negatively correlated with the miR-200a-3p. **E** and **F** Upregulation of miR-200a-3p increases the sensitization of osteosarcoma cells to MTX, while upregulation of OGT almost abolished the effect of miR-200a-3p. *p < 0.05, **p < 0.01, compared with miR-200a-3p mimic; ^#^*p* < 0.05, ^##^*p* < 0.01, compared with miR-200a-3p mimic + OGT OE

To further identify the targeted regulating gene of miR-200a-3p, the TargetScan was used to search for the target gene. The O-GlcNAc transferase (OGT), a proven elevated protein in many tumors [23], was found to be one of the binding genes of miR-200a-3p (Fig. 4A). The immunohistochemistry and western analysis showed that increased expression of OGT is obvious when compared to adjacent tissues (Fig. 4B, C). We observed a negative relationship between miR-200a-3p and OGT expression levels in osteosarcoma tissues (Fig. 4D). Furthermore, upregulation of OGT almost counteracts the sensitization effect of miR-200a-3p to MTX in osteosarcoma cells (Fig. 4E, F).

### EBLN3P increased MTX-resistance of osteosarcoma cells by enhancing miR-200a-3p/OGT axis

**Fig. 5:**
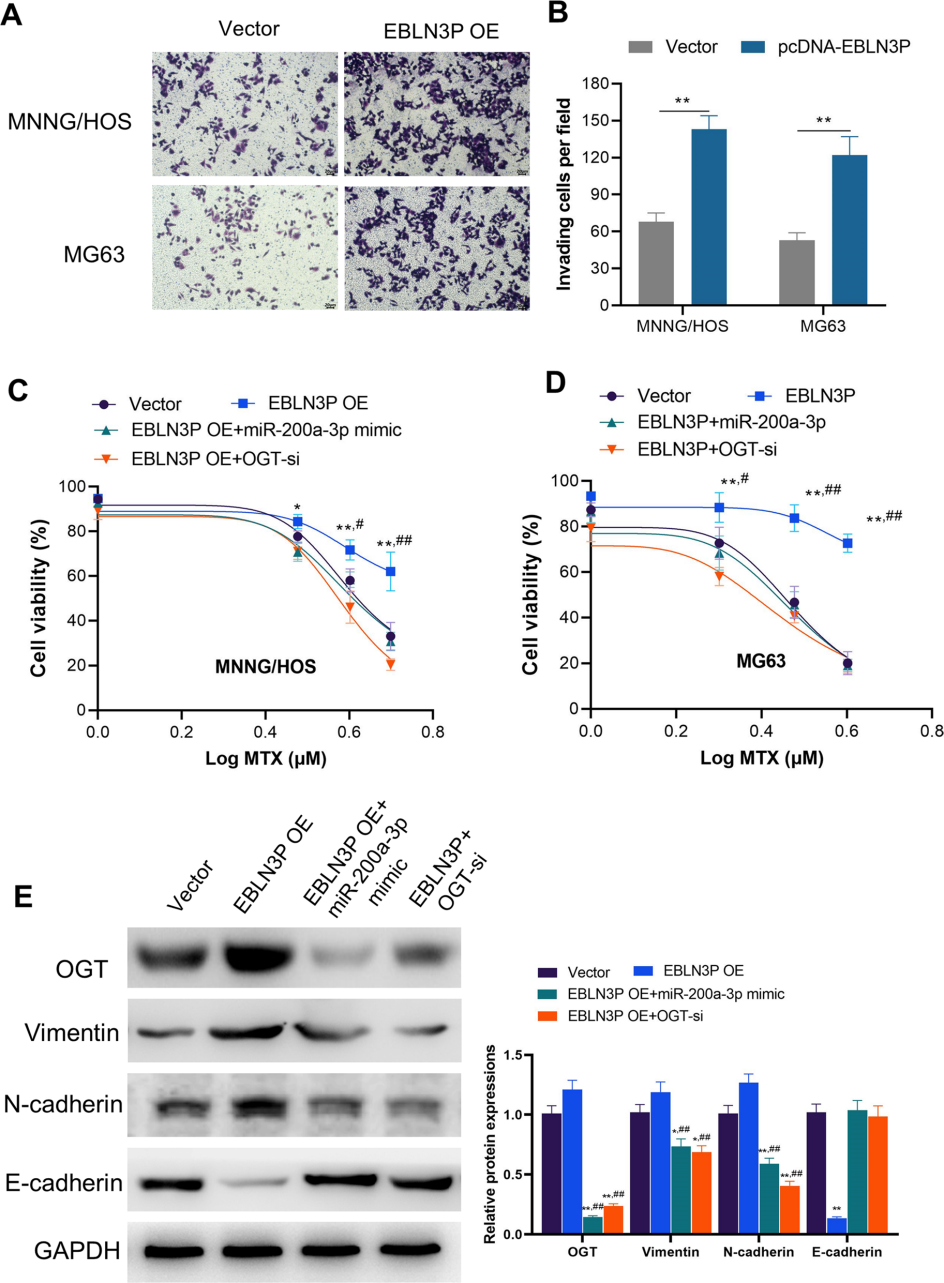
EBLN3P increased MTX-resistance of osteosarcoma cells by enhancing miR-200a-3p/OGT axis. **A** Upregulation of EBLN3P (EBLN3P OE) increased the invasion ability of MNNG/HOS and MG63 cells. **B** Column comparison of invaded cells in MNNG/HOS and MG63 cells. **p < 0.01. **C** and **D** Effect of blocking OGT on EBLN3P upregulated cells was evaluated. Blocking OGT by OGT-si decreased the resistant effect of EBLN3P on MTX in MNNG/HOS and MG63 cells. *p < 0.05, **p < 0.01, compared with vector; ^#^*p* < 0.05, ^##^*p* < 0.01, compared with EBLN3P OE + OGT-si. **E** Western blot analyzing the effect of EBLN3P, miR-200a-3p, and OGT on MNNG/HOS cells. Upregulation of EBLN3P leads to increased expression of OGT, Vimentin, and N-cadherin while decreasing the expression of E-cadherin. Further upregulation of miR-200a-3p or knockdown OGT (OGT-si) decreased the expression of OGT, Vimentin, and N-cadherin. *p < 0.05, **p < 0.01, compared with vector; ^#^*p* < 0.05, ^##^*p* < 0.01, compared with EBLN3P OE

The effect of EBLN3P on osteosarcoma cells was measured through transfecting pcDNA- EBLN3P to osteosarcoma MNNG/HOS and MG63 cells. We observed that upregulation of EBLN3P increased the invasion and MTX resistance of both cells (Fig. 5A–D). Blocking OGT by OGT-si crippled the resistant effect of EBLN3P on MTX in osteosarcoma cells (Fig. 5C, D). Moreover, miR-200a-3p could not increase the sensitivity of MTX in osteosarcoma cells after OGT was overexpressed (Fig. 5C, D). Research has demonstrated that OGT promotes EMT and invasion of cancer cells mainly via the junction with skeleton proteins [24–26]. We further detected the expression of invasion markers of EMT under the influence of loss-of-function of OGT. Western blot analysis also demonstrated that upregulation of EBLN3P increased protein expression of OGT, Vimentin, and N-cadherin while the expression of E-cadherin decreased (Fig. 5E).

## Discussion

Osteosarcoma is highly malignant and tends to metastasize at the early stage. For patients with osteosarcoma, most current treatment programs usually involve intensive neoadjuvant chemotherapy [27]. However, chemotherapy resistance is an important factor affecting the disease progression and survival of patients with osteosarcoma [28]. Our study shows that EBLN3P can affect the MTX resistance of osteosarcoma cells by regulating miR-200a-3p, an important microRNA that affects EMT. Reversing chemotherapy resistance is of great significance to cancer management strategies. The mechanisms of drug resistance in tumors are complex and diverse, which are mainly related to ABC transporter, Topo II, oncogenes Bcl-2, p53, NF-κB, and other factors [29, 30]. MDR1 (P-gp) is one of the main causes of drug resistance in a variety of tumors, including osteosarcoma [31, 32]. It can expel chemotherapeutic drugs out of cells against the concentration gradient, thereby increasing the tolerance of tumor cells to chemotherapeutic drugs. The ability of tumors to reduce the uptake of drug molecules is also considered to be a mechanism of drug resistance. This mechanism is similar to the way of increasing drug efflux (or drug transport to intracellular concentration decreased), which will reduce the concentration of drug molecules in the cellular environment, thereby limiting its efficacy on target tumors. The chemotherapy drug molecules are most susceptible to this resistance mechanism, such as MTX, 5-fluorouracil, 8-azaguanine, and cisplatin. They have been proved to use transporters such as solute carriers (SLCs) to enter cells [33, 34].

MTX is an anti-folate cellular immunosuppressant. It mainly inhibits purine synthesis by blocking dihydrofolate reductase, thus inhibiting thymine synthesis, reducing the chemotaxis of neutrophils, and inhibiting the release of inflammatory cytokines. In osteosarcoma cells, MTX binds to the dihydrofolate reductase (DHFR) enzyme, leading to inhibition of DNA synthesis and replication and ultimately causing apoptosis [33], which induced apoptosis or programmed cell death due to induced DNA damage [35]. As a response, some cell lines can form clonal expansion colonies, express DNA repair enzymes, and produce strong resistance to MTX, cisplatin, and other drugs. The drug resistance of MTX in osteosarcoma is related to the above factors in different ways, we speculate there might be genes involved in MTX transport and metabolism might be regulated by genes of the miR-200a-3p/OGT axis.

EBLN3P is a novel lncRNA, reports show that expression of EBLN3P is upregulated in a variety of tumors. Existing research evidence shows that it plays as oncogene in tumor cells through different miRNA/target gene axes [10, 13]. Our study shows that EBLN3P promotes the metastasis and drug resistance of osteosarcoma through miR-200a-3p/OGT axis. The miRNAs also are closely related to the occurrence of bone-related diseases [8, 9, 36]. Acted as effective regulatory genes, miRNAs have been demonstrated binding their target gene such as ERK 2, TGF- β1, and Smad3 to regulate tendon homeostasis [36]. miR-200a-3p is a member of the miR-200 family, and this family controls EMT process both in development and tumorigenesis [37]. miR-200 also mediates the chemoresistance of tumor cells. Pan et al. [38] reported that miR-200b, a member of the miR-200 family regulates autophagy associated with chemoresistance in human lung adenocarcinoma. This is consistent with Jin’s report [39] that downregulated miR-200b is involved in the chemoresistance process. Same as miR-200b, miR-200a also act as a tumor-suppressive gene in hepatocellular carcinoma [40]. Our study showed that miR-200a-3p was downregulated in osteosarcoma, resulting in the downregulation of downstream target gene OGT, which in turn promoted the EMT process of osteosarcoma cells, leading to the metastasis and MTX resistance of osteosarcoma cell.

EMT is a key mechanism of tissue remodeling during multicellular morphogenesis. The role of EMT in drug resistance has been confirmed [41]. Loss of the epithelial phenotype is the most significant feature of EMT; meanwhile, the cells obtained the interstitial phenotype, and the expressions of vimentin, N-cadherin increased. O-GlcNAc glycosylation modification is widely involved in many cells’ life processes, such as cell cycle, gene transcription, protein translation and processing, and signal transduction. Abnormal modification of O-GlcNAc glycosylation occurred in many diseases [42]. Studies have confirmed that OGT is highly expressed in liver tumors [43], colon tumors, bladder cancer, and breast cancers [44].

Inconsistent with the reported results of Sombutthaweesri et al. [45], our study showed that the expression of OGT was increased in surgical resection samples of osteosarcoma, and the upregulated expression was associated with MTX resistance. We speculate that inconsistency of OGT expression might be related to different clinical characteristics of patients (stage, age, course of disease, and background treatment). Moreover, our study also showed that decreasing the expression of OGT in osteosarcoma cells could significantly enhance the sensitivity of osteosarcoma cells to MTX. In tumor cells, O-GlcNAc proteins could promote vimentin expression and cell migration [46], while OGT catalyzes O-GlcNAc proteins to exert its effect [47]. Chen et al. demonstrated that OGT could restrain the expansion of DNA damage [48]. Moreover, Barkovskaya et al. [49] reported that inhibition of OGT could activate the expression of the tumor-suppressor gene in tamoxifen-resistant breast cancer cells. These results indicate that OGT contributes the drug resistance in some tumors. By repairing damaged DNA and increasing the EMT of tumor cells, tumor cells strengthen the discharge of these chemotherapy drugs and increase the resistance to these agents.

Our study has several limitations. First, the number of tumor samples involved in the current study is small, and tumor tissues from more patients are further needed to confirm the relationship between LncEBLN3P with clinical parameters. Second, the major hypothesis in the present research has not been verified by in vivo research, so the current conclusion is preliminary. In vivo investigation and deeper clinical research are necessary to explore how does LncEBLN3P/miR-200a-3p/OGT axis regulate the MTX sensitivity in osteosarcoma.

In conclusion, our research demonstrated that LncEBLN3P is upregulated in osteosarcoma and affects the MTX resistance in osteosarcoma cells through downregulating miR-200a-3p, which in turn promoted the EMT process of osteosarcoma cells by increased OGT, and this needs to be further confirmed in the follow-up study.
